# Particle removal from air by face masks made from Sterilization Wraps: Effectiveness and Reusability

**DOI:** 10.1371/journal.pone.0240398

**Published:** 2020-10-14

**Authors:** Sachin Walawalkar, Manish Joshi, Navin Khattry, Balvinder Kaur Sapra, Arshad Khan, Pradeep Kumar Pujari, Lalit Mohan, Sushil Prasad Srivastava, Chital Naresh, Rajendra Badwe, Sudeep Gupta

**Affiliations:** 1 Advanced Centre for Treatment, Research and Education in Cancer (ACTREC), Tata Memorial Centre, Navi- Mumbai, India; 2 Radiological Physics and Advisory Division, Bhabha Atomic Research Centre (BARC), Mumbai, India; 3 Homi Bhabha National Institute, Mumbai, India; 4 Radiation Chemistry and Isotope Group, Bhabha Atomic Research Centre (BARC), Mumbai, India; 5 Centre for Design and Manufacture (CDM), Bhabha Atomic Research Centre (BARC), Mumbai, India; VIT University, INDIA

## Abstract

Wearing face masks is highly recommended to prevent SARS-CoV-2 transmission in health care workers and for the general public. The demand for high quality face masks has seen an upsurge in the recent times, leading to exploration of alternative economic and easily available options, without compromising on the quality. Particle removal from air in terms of capture efficiency of the filter media or the face mask is a crucial parameter for testing and quality assurance. Short-term reusability of the face masks is also an important aspect as the demand for masks will potentially outstrip the supply in future. Sterilization Wraps, which are used to wrap sterile surgical instruments, have shown a promising performance in terms of removal of particles from air. In this study, we evaluate the particle filtration characteristics of face masks made of 2 different metric weights [45 and 60 gram per square metre (GSM)] respectively, using locally available Sterilization Wraps. The aerosol filtration characteristics were also studied after sterilisation by different techniques such as heat with 50% humidity (thermal treatment), ethylene oxide (ETO), steam and radiation dose of 30kGy. We found that 60 GSM face mask had particle capture efficiency of 94% for total particles greater than 0.3 microns and this capture efficiency was maintained even after sterilisation with ETO and thermal treatment. The cost of producing these masks was 30 US cents/mask at our institute. Our study suggests that sterilization wrap material made of non-woven polypropylene spunbond-meltblown-spunbond (SMS) fibres could be an appropriate readily available inexpensive material for making face masks or N95 respirators.

## Introduction

As the mode of transmission for SARS-CoV-2 includes droplets, aerosols and physical contact [1], usage of face mask and social distancing are recommended as crucial strategies for preventing infection [2]. Face masks are designed and intended to reduce the inhalable concentration of particulates, when worn properly by the user. Different types, designs and grades of face masks are available for use by infected persons, health care workers and general public. The characteristics of face masks vary and a choice is made based on the availability, risk profile and suggested guidelines. For the general public, a shortage of face masks can be countered by the usage of alternatives such as filters made up of common and uncommon fabric materials [3–5]. This strategy is substantiated by the current understanding on the transmission characteristics of SARS-COV-2 pathogens [6]. The particle removal efficacy for the cloth filters and common fabrics has been shown to be reasonably sufficient when challenged to the particles of sizes more than 1 μm [7]. However, in healthcare environments, the possibility of pathogen loading in sub-micrometer size ranges during treatment procedures [8] and properties such as splash resistance and blood penetration ability [9], have posed limitations on the type of face masks that can be used in such environments. Any shortage in supplies can only be compensated by an equivalent alternative, with similar desired characteristics as per the guidelines.

Recently, “Sterilization Wraps” (SW) have been evaluated as one such option owing to their wide spread availability and aerosol filtration characteristics [10–12]. Sterilization Wraps are used to wrap surgical instruments which after sterilization procedures form a formidable barrier to entry of micro-organisms. These wraps are usually made of non-woven polypropylene synthetic fabric consisting of spunbond-meltblown-spunbond (SMS) fibres. These fibres form a matrix that prevents micro-organisms from penetrating the inner layer of the sterilization wrap. Studies have also shown their benefit in terms of fluid resistance characteristics and possibility of reuse [10] after due sterilization procedures. Apart from the conventional sterilization methods used for surgical wraps, viz. ethylene oxide (ETO) and steam treatment, it would be interesting to measure and interpret the particle removal characteristics when these wraps are treated to heat with appropriate relative humidity (Thermal Treatment) or ionising radiation. Whereas thermal sterilization is simple and an effective way to decontaminate pathogenic load [13], ionising radiation can also act as a viable option supplementing large scale decontamination strategies. In the past, exposure of face masks to radiation dose (10–30 kGy) has been shown to degrade the performance of standard N-95 respirators [14]. The objective of our study was to measure the capture efficiency of aerosol particles for face masks made from Sterilization Wraps in pre- and post- sterilized conditions. If found to efficacious, face masks manufactured from this material would be an easily available inexpensive alternative to those made from commercially available standard filtration media.

## Material and methods

### Face mask configured from Sterilization Wraps

**Fig 1:**
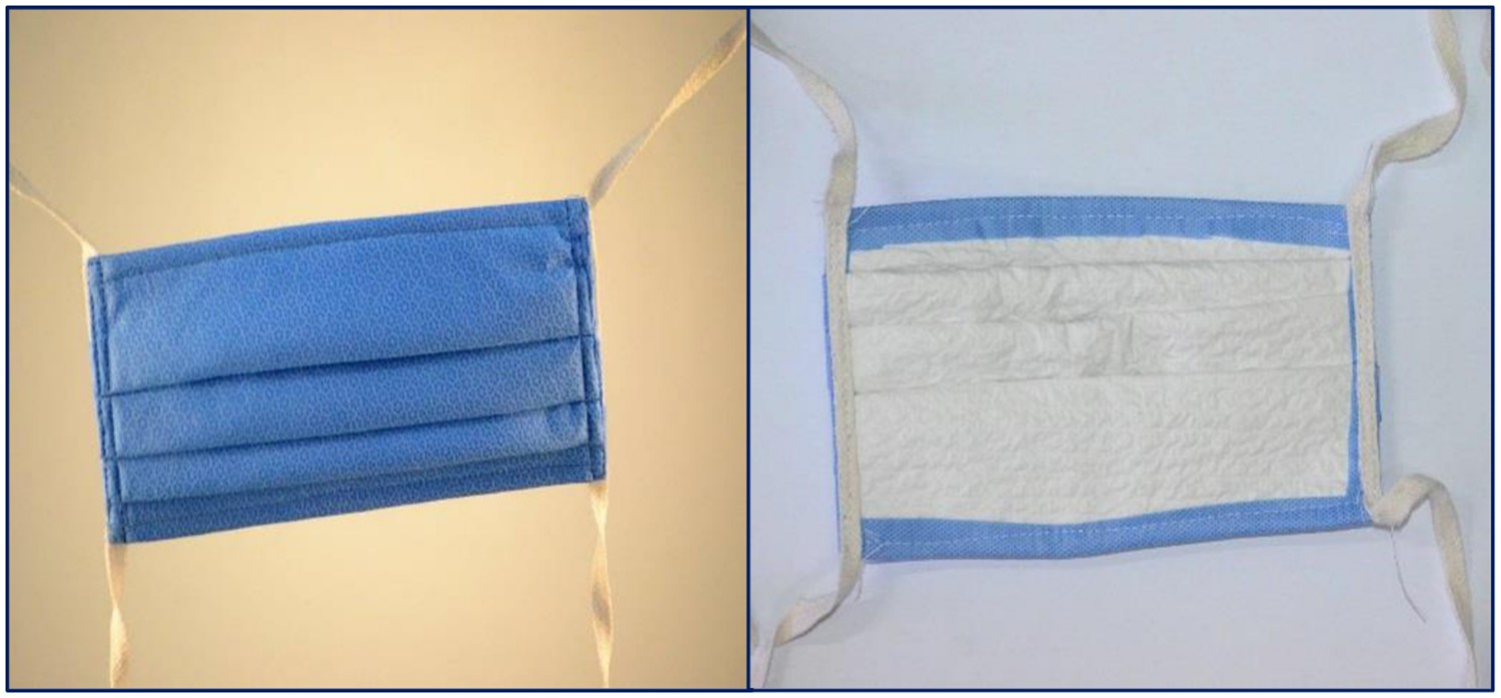
Face mask derived from 60 GSM fabric.

We used sequential sterile wrap, made from Kimguard fabric technology (H300), of 2 different metric weights [45 and 60 gram per square metre (GSM)] respectively, to configure locally made face masks. Each mask comprised 2 layers of 45 GSM or 60 GSM, machine stitched to form the outer layer, with the inner layer formed by 2 layers of cellulose hand wipe sheets (Fig 1). The inner layer absorbs moisture during breathing to keep the wearer comfortable while the outer layer removes the particles from the air inhaled by the wearer. The masks used during the experiments were in square shapes of dimensions 8 cm x 8 cm. These masks were tested for their aerosol capture efficiency both at baseline condition and post-sterilization. Sterilization techniques used included 1 cycle of ethylene oxide (ETO) treatment at 37°C, steam exposure at 121°C, heat treatment at 69°C with 50% relative humidity for 30 minutes (Thermal Treatment) and radiation exposure with 30 kGy gamma dose, respectively.

### Set-up and instrumentation for particle capture efficiency

Particle capture efficiency for face masks configured from both 45 and 60 GSM sterilization wrap fabrics was measured in tests performed in an ‘intrinsic sampler’. We also measured the capture efficiency of a commercially available N 95 respirator and surgical face mask for representative purpose. This sampler was made up of a cylindrical glass pipe of volume 2.2 liters (140 cm length and 4.5 cm diameter) wherein aerosol/auxiliary instrumentation can be connected. The face mask material was cut into circular samples of 4.5 cm diameter and affixed in the line of air flow using clamping arrangements. A commercial aerosol atomizer (model no TOPAS ATM226) was employed for generating NaCl test particles for the tests performed in this study. Particles generated from the generator were dried, charge neutralized and mixed in a 40 litre Perspex mixing chamber before passing through the intrinsic sampler. An optical particle counter (OPC) was used for the measurements of number concentration of the test particles. We used Grimm OPC (model no. 1.108) for number size distribution measurements in the size range of 0.3–20 μm. It was operated at the flow rate of 1.2 Lmin^-1^ and sampled particles from the ‘IN’ and ‘OUT’ ports provided in the intrinsic sampler. These ports sampled number concentration of NaCl particles upstream and downstream of the test specimen in steady state conditions. More details on the set-up, instrumentation and methodology can be found elsewhere [15]. The set-up used during these experiments, flow rate (28.3 Lmin^-1^) and corresponding flow velocity (29.67 cm/sec) conformed to the testing procedure given in standard codes [16, 17]. The test parameter i.e. aerosol capture efficiency (η_i_) of the filter material was defined as the ratio of captured to incident particles. It was estimated from the number concentrations measured at ‘IN’ and ‘OUT’ port signifying upstream (C_upstream_) and downstream (C_downstream_) number concentration of the test particles. The equation was written as follows:

$$\eta_{i}=\frac{C_{upstream}-C_{downstream}}{C_{upstream}}$$ (1)

All tests were repeated 5 times and error propagation rules were used for reporting the mean values and the standard deviation (1σ).

## Results and discussion

The first objective of the test experiments pertains to the measurement of capture efficiency of 45 GSM and 60 GSM prototype masks for different particle sizes. Table 1 shows the results of these measurements for three particle sizes (0.3, 0.5 and 1 μm) and the entire size range, respectively. The stated particle sizes are the lower end of the size range corresponding to different size channels of OPC. For comparison, results from similar test measurements performed with representative commercial N-95 respirator and surgical mask are also given in this table. These comparisons were made for the representative cut samples (filter material) of the face masks/respirators. In addition to the performance of the media, fit factor of the mask/respirator is a crucial parameter which determines the final performance of the mask/respirator.

**Table 1:** Aerosol capture efficiency for test specimens.

S. No.	Mask type (n = 5)	Aerosol capture Efficiency (%) for particle size			
		0.3 μm	0.3 μm	0.3 μm	0.3 μm
1.	45 GSM SW	82.65±3.08	88.61±4.29	94.01±8.57	84.88±3.50
2.	60 GSM SW	92.10±3.69	98.19±5.64	99.13±7.44	94.48±4.18
3.	Surgical mask(commercial)	40.08±3.85	56.35±4.41	84.12±6.76	42.44±3.91
4.	N-95 respirator (commercial)	96.19±2.55	96.67±2.18	100.00±0.00	96.64±2.21

As seen in Table 1, the aerosol capture efficiency measured for the entire size range (>0.3 μm) for 45 GSM and 60 GSM Sterilization Wraps were 84.88±3.50% and 94.48±4.18%, respectively. The values measured for these specimens were significantly higher than the commercially available tested surgical mask and appreciably closer to those of a commercially available N-95 respirator tested in the above experiments.

**Fig 2:**
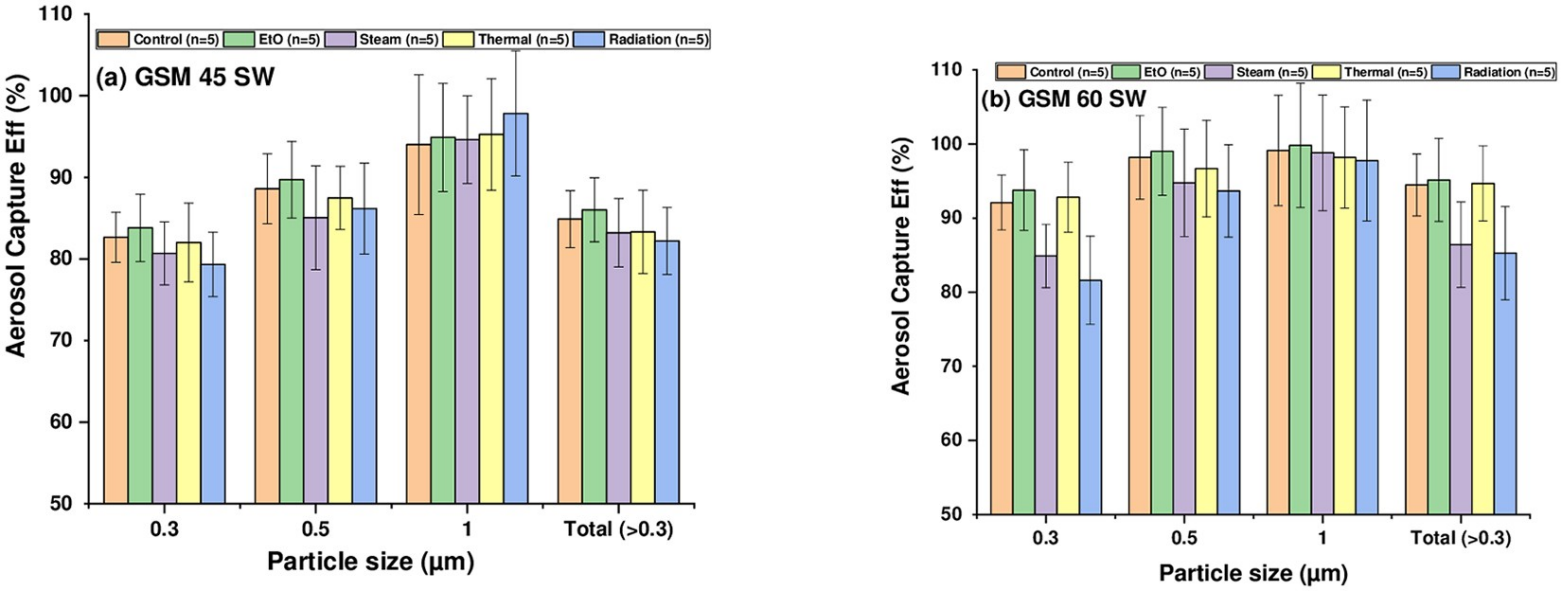
Aerosol capture efficiencies for GSM 45 and GSM 60 surgical wrap face mask.

In the next step, aerosol capture efficiencies were measured for the control (untreated) and sterilized (treated) samples of 45 and 60 GSM Sterilization Wraps employing different sterilization processes as mentioned earlier. Fig 2 shows the effect of one cycle of sterilization treatment (ETO, Steam, Thermal Treatment and Radiation respectively) on the capture efficiencies measured for masks configured of 45 and 60 GSM Sterilization Wraps.

It can be inferred from Fig 2 that aerosol capture efficiency remained similar after sterilization with different methods for 45 GSM SW face mask. As far as sterilization of 60 GSM SW face mask was concerned, decrease in efficiency post sterilization was noted for steam and radiation treatment while there was no drop in capture efficiency with ETO and thermal treatment as shown in Fig 2. However, the 60 GSM-filter had a higher capture efficiency for 0.3 μm particles as compared to that of 45 GSM filter, both in untreated and after sterilization with ETO and Thermal Treatment. respectively. In addition to the above favourable properties, these masks were manufactured at a very low cost i.e. 30 US cents/masks.

## Conclusions

The first level impressions indicate that 60 GSM SW face mask l made of non-woven polypropylene SMS fibres could be an appropriate readily available substitute to high quality commercially available face masks/respirators. The affordability in terms of cost and the potential benefit for short term re-usage further enhances its utility.

## Supporting information

S1 Data (XLSX)
