# Correction: Particle removal from air by face masks made from Sterilization Wraps: Effectiveness and Reusability

**DOI:** 10.1371/journal.pone.0252693

**Published:** 2021-05-27

**Authors:** Sachin Walawalkar, Manish Joshi, Navin Khattry, Balvinder Kaur Sapra, Arshad Khan, Pradeep Kumar Pujari, Lalit Mohan, Sushil Prasad Srivastava, Chital Naresh, Rajendra Badwe, Sudeep Gupta

There is an error in Table 1. In the second row, the values in columns four, five, and six are incorrect. Please see the correct Table 1 here.

**Table 1:** Aerosol capture efficiency for test specimens.

S. No.	Mask type (n = 5)	Aerosol capture Efficiency (%) for particle size			
		0.3 μm	0.5 μm	1.0 μm	Total (>0.3 μm)
**1.**	**45 GSM SW**	**82.65±3.08**	**88.61±4.29**	**94.01±8.57**	**84.88±3.50**
**2.**	**60 GSM SW**	**92.10±3.69**	**98.19±5.64**	**99.13±7.44**	**94.48±4.18**
**3.**	**Surgical mask (commercial)**	**40.08±3.85**	**56.35±4.41**	**84.12±6.76**	**42.44±3.91**
**4.**	**N-95 respirator (commercial)**	**96.19±2.55**	**96.67±2.18**	**100.00±0.00**	**96.64±2.21**
